# Preharvest Application of Oxalic Acid to ‘Calabacita’ Fresh Figs: Effects on Physicochemical and Antioxidant Profile During Cold Storage

**DOI:** 10.3390/foods14234061

**Published:** 2025-11-27

**Authors:** Carlos Moraga-Lozano, Ana María Fernández-León, Margarita López-Corrales, Alicia Rodríguez, Manuel J. Serradilla, Mónica Palomino-Vasco

**Affiliations:** 1 Centre for Scientific and Technological Research of Extremadura (CICYTEX), Department of Postharvest, Plant Value Enhancement, and Emerging Technologies, Junta de Extremadura, Avda. Adolfo Suárez s/n, 06007 Badajoz, Spain; carlos.moraga@juntaex.es (C.M.-L.); anamaria.fernandezl@juntaex.es (A.M.F.-L.); monica.palomino@juntaex.es (M.P.-V.); 2 Centre for Scientific and Technological Research of Extremadura (CICYTEX), Department of Mediterranean Fruticulture, Junta de Extremadura, A-5, km 372, 06187 Guadajira, Spain; margarita.lopez@juntaex.es; 3 University Institute for Agricultural Resources (INURA), Department of Animal Production and Food Science, University of Extremadura, Avda. Adolfo Suárez s/n, 06007 Badajoz, Spain; aliciarj@unex.es

**Keywords:** *Ficus carica* L., elicitor, quality, bioactive compounds

## Abstract

Fresh figs are a highly perishable fruit with a very limited shelf life. Consequently, the development of innovative strategies at both the preharvest and postharvest stages is essential to enhance their quality and extend their shelf life. This study aimed to evaluate the postharvest performance of fresh figs (cv. Calabacita) treated preharvest with oxalic acid (OA) via foliar spraying at 1.2 L per tree at two concentrations (1 and 2 mM), applied either twice or three times. Figs were harvested at commercial maturity and stored for 10 days at 1 °C and 90% relative humidity in darkness, with sampling carried out at 0, 3, 7 and 10 days. At each sampling point, physiological, physicochemical, and bioactive parameters were assessed, and an analysis of variance was performed to determine differences among OA treatments. The findings showed that the effectiveness of OA depended on the number of applications, with two preharvest sprays providing the most favourable outcomes. OA at 2 mM significantly reduced weight loss, respiration rate, and ethylene production compared with controls and increased titratable acidity. Furthermore, all OA treatments enhanced the antioxidant activity of the fruit, improving both enzymatic and non-enzymatic antioxidant activity, as well as total phenolic content. This suggests improved stress tolerance supported by lower cell wall oxidation at the end of cold storage. In conclusion, two preharvest applications of oxalic acid effectively contribute to maintaining fruit quality and extending the storability of fresh figs during cold storage.

## 1. Introduction

The fig is the fruit of *Ficus carica* L., a species belonging to the Moraceae family that produces an inflorescence with a distinctive syconium structure. This species adapts well to diverse soil types and thrives in mild to subtropical climates. It holds considerable agronomic significance worldwide, as it can be consumed either fresh or dried, with or without skin [1,2,3,4]. Production is mainly concentrated in the Mediterranean region [1,3,5,6,7], with Spain being the leading European producer [8].

Figs are widely recognised as a health-promoting fruit with high nutritional value and medicinal properties and have been employed in the treatment of a broad range of diseases (i.e., gastrointestinal, inflammatory, or metabolic conditions, among others) [9]. They are characterised by a rich composition of sugars, organic acids, vitamins, minerals, proteins, and amino acids. Figs have also been shown to provide significant amounts of antioxidants, such as phenolic acids and flavonoids, which exhibit anti-inflammatory and antibacterial activity [1,2,3,5,6,8,10,11]. These compounds not only contribute to human health but also play a key role in determining the colour, flavour, and aroma of the fruit, as well as its overall organoleptic quality [11,12]. The nutritional and bioactive traits of figs are influenced by numerous factors, including genotype, soil, climatic conditions, agronomic practices, and ripening stage [4,6,7,8]. Furthermore, it should be noted that the consumer acceptance of figs is strongly affected by morphological and sensory attributes, particularly colour, aroma, and firmness [10].

Nevertheless, the main challenge associated with fresh figs is their limited storability, which typically ranges from 5 to 7 days in cold storage. This is mainly due to their high perishability [4,7,8], which can be attributed to the fruit’s thin skin, rapid respiratory rate, and the natural opening known as the ostiole, which facilitates the entry of microorganisms such as moulds of the genera *Alternaria*, Cladosporium and Penicillium [11,13]. During storage, various physiological and physicochemical changes occur as a result of both internal and external factors, primarily oxidative stress, leading to quality losses partly through the generation of reactive oxygen and nitrogen species [14]. Consequently, there is a clear necessity to develop diverse pre- or postharvest strategies aimed at extending the shelf life of fresh figs.

To achieve the aforementioned objective and reduce food waste effectively, it is essential to implement strategies aligned with sustainability principles. Bearing that in mind, the European Green Deal, together with the Farm to Fork initiative, was created to attempt to reduce the use of pesticides by at least 50% by 2030 [15].

One of the most widely adopted approaches for enhancing fruit quality traits and reducing postharvest losses is the use of elicitors and other compounds such as salicylic acid [16], 1-methylcyclopropene [17], abscisic acid [18], and calcium chloride [19]. Among these, one of the best known is oxalic acid (OA), an organic acid that occurs naturally in plants. OA has been shown to modulate physiological processes involved in on-tree fruit development, as demonstrated in cherries. On-tree fruit development was influenced by preharvest OA treatment by increasing fruit size and weight, as well as other parameters such as total anthocyanins, total phenolics, and antioxidant activity [20]. Several studies have demonstrated its significant role in systemic acquired resistance and environmental responses. The application of OA, either pre- or postharvest, has been shown to enhance physicochemical traits and to extend shelf life [21,22]. This effect is mainly attributable to the slowing of respiration and ethylene production rates, together with an increase in the content of bioactive compounds and both enzymatic and non-enzymatic antioxidant activity. Consequently, OA reduces browning and decay in various horticultural crops, such as apricot and kiwi, from 1 to 10 mM of OA concentration [14,23,24,25]. Although the precise mechanism by which OA enhances the antioxidant capacity of fruits is not yet fully understood, its protective efficacy may be related to both its inherent antioxidant activities and its role in the regulation of ethylene signalling [14,23,26].

It is noteworthy that the effects of preharvest elicitors are dependent on their concentration, the number of applications, and the timing of application for each crop [27]. Despite the widespread use of OA as a preharvest treatment to enhance the traits of various fruits, to the best of our knowledge, no study has yet evaluated its effect on fresh figs. Therefore, the aim of this study was to assess the effect of preharvest OA treatments, applied at two concentrations (1 and 2 mM) and sprayed either two or three times, on the physiological, physicochemical, and bioactive traits of fresh, green-skinned figs during cold storage.

## 2. Materials and Methods

### 2.1. Plant Material and Experimental Design

The present study was conducted in 2023 in an experimental orchard of fig trees (*Ficus carica*, L.; cv. Calabacita) located at the Centre for Scientific and Technological Research of Extremadura (CICYTEX) (‘Finca La Orden’; latitude 38°85′19″ N, longitude −6°68′28″ W; Guadajira, Badajoz, Spain). The trees, planted in 2019, were derived from cuttings of ‘Calabacita’ trees located in the National Fig Germplasm Bank at CICYTEX. These trees were trained in an espalier system within a high-density planting with a spacing of 3 × 2.5 m and were subjected to standard agronomic practices. For each treatment, three blocks of four randomly selected trees were used.

Treatments were oxalic acid (Sigma-Aldrich, St. Louis, MO, USA) 1 (OA1) and 2 mM (OA2), along with a water-treated control (CO). All treatments included 0.2 mL L^−1^ Tween 20 (Chemsolute, Th. Geyer GmbH & Co. KG, Renningen, Germany) as a surfactant and were prepared using tap water. To evaluate the effect of the number of applications, two and three applications of the different treatments were performed. The OA concentrations were selected based on previous studies on other climacteric fruits [28,29]. The number of application assessments was carried out due to staggered ripening on the same branch [30]. The first application was carried out when most of the figs were transitioning from stage II to III (diameter between 30 and 35 mm), once they had reached physiological maturity. The end of Phase II is a key moment in the ripening of figs because at this stage, there is an increase in the respiration rate and ethylene production [31]. Furthermore, Phase III is the ripening phase, which includes fruit growth, colour change, softening and alteration of the pulp firmness to an edible state [32]. The interval between applications was one week. All applications were made by foliar spraying, using 1.2 L of solution per tree, and applied early in the morning.

According to previous research from our group [12,33], figs were harvested at commercial maturity (diameter between 40 and 45 mm) and cold-stored at 1 ± 0.5 °C and 90 ± 5% relative humidity in standard atmosphere and in darkness. Sampling was carried out at harvest (day 0) and after 3, 7, and 10 days of storage.

### 2.2. Methodology

The physiological stage of figs was determined by measuring respiration and ethylene production rates (three replicates of 10 fruits each; n = 3). Respiration rate (mL CO_2_ kg^−1^ h^−1^) was determined in a static system using a Checkpoint 3 gas analyser (PBI Dansensor, Barcelona, Spain). Ethylene production was also measured in a static system using a GC-7890A gas chromatograph (Agilent Technologies, Santa Clara, CA, USA) and results were expressed in µL C_2_H_4_ kg^−1^ h^−1^.

Regarding physicochemical parameters, 30 fruits per treatment and sampling day were randomly selected. Fruit weight was recorded in grams (g) using a CB Complet electronic balance (Cobos, Barcelona, Spain). Weight loss during cold storage was calculated as the percentage (%) difference between the initial weight and the weight on each sampling day. Firmness was evaluated using a TA.XT2i Texture Analyser (Stable Micro Systems, Godalming, UK) on two opposite faces of each fig. The instrument was equipped with a 25 mm flat-base probe (test speed of 0.2 mm s^−1^), and results (N mm^−1^) were expressed as the ratio between the force required to produce a 6% deformation and the fruit size. Colour was measured on two opposite faces of each fig, for both skin and flesh, using CIELab space coordinates to obtain luminosity (L*), chroma (C*) and hue angle (h*), with a Chroma Meter CR-400 tristimulus colorimeter (Konica Minolta, Valencia, Spain).

Once the non-destructive analyses were completed, the 30 fruits from each treatment and sampling day were divided into three independent replicates (10 fruits per replicate, n = 3), which were chopped and homogenised. Total soluble solids (TSS) were measured using a PR-01 digital refractometer (Atago Co., Ltd., Tokyo, Japan), and results were expressed in °Brix. Titratable acidity (TA) was determined using an automatic titrator T50 Graphix (Mettler Toledo, Madrid, Spain) with NaOH 0.1 N, and results were expressed as % citric acid. The ripening index (RI) was calculated as the TSS/TA ratio. Finally, sugars and organic acids were identified and quantified chromatographically as described by Serradilla et al. [34], using 1 g of sample extracted with water for 1 h. Results were expressed as g kg^−1^ FW (fresh weight).

Antioxidant activity was assessed both non-enzymatically and enzymatically. Total non-enzymatic antioxidant activity (TAA) was measured using the DPPH and ABTS methods, following the methodologies of Pérez-Jiménez et al. [35], with a UV-2401PC spectrophotometer (Shimadzu, Kyoto, Japan) and results were expressed in mg Trolox equivalent 100 g^−1^ FW. Total phenolic content (TPC) was determined using the Folin–Ciocalteu colourimetric method; results were expressed in mg gallic acid equivalent 100 g^−1^ FW. Enzymatic antioxidant activity was assessed by measuring the activities of ascorbate peroxidase (APX), catalase (CAT) and peroxidase (POD), following the protocol of Carrión-Antolí et al. [36] using a UV-2401PC spectrophotometer (Shimadzu, Kyoto, Japan). Results were expressed as U min^−1^ g^−1^, where U represents enzyme activity units. Finally, lipid oxidation of cell membranes was evaluated by spectrophotometric quantification of malondialdehyde (MDA), according to the method described by Bi et al. [37]. All determinations were carried out in triplicate (samples of 10 fruits; n = 3).

### 2.3. Statistical Analysis

The experiment was arranged as a randomised complete block design (RCBD), with blocks included to account for potential spatial variability within the orchard. Within each block, the different oxalic acid treatments were randomly assigned to the experimental units. Data are presented as the mean ± standard deviation. The datasets for two and three applications were analysed separately, comparing the effect of the different treatments on each sampling day. For each parameter, the results for each sampling day and number of applications were subjected to a one-way analysis of variance (ANOVA) using SPSS 25.0 for Windows (IBM, Armonk, NY, USA). When significant differences were found, Tukey’s test was used for multiple comparison of means. Differences were considered significant at *p* < 0.05.

## 3. Results

### 3.1. Physiological Parameters

**Figure 1:**
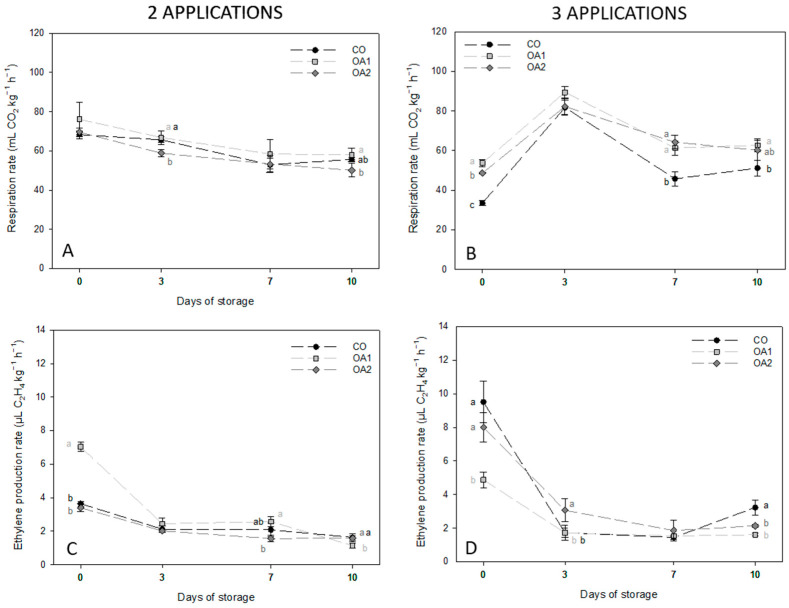
Mean values ± standard deviation of respiration (**A**,**B**) and ethylene production (**C**,**D**) rates in fruits treated with two and three applications, respectively, across the four sampling dates. Different lowercase letters within each day indicate significant differences (*p* < 0.05) according to Tukey’s test. CO: control; OA1: oxalic acid 1 mM; OA2: oxalic acid 2 mM.

Respiration and ethylene rate data are presented in Figure 1. Considering respiration first, fruits treated with two applications (Figure 1A) showed a decrease in respiration rate during storage. Significant differences between treatments were observed only on days 3 and 10, where the OA2-treated figs showed lower values than the control and OA1 treatments. In fruits treated with three applications (Figure 1B), by contrast, respiration peaked on day 3, while values on the remaining days remained relatively stable. In this case, significant differences were found between the CO and treated fruits, with the latter showing higher values on all sampling dates.

Regarding ethylene production, the trend was similar for fruits treated with two (Figure 1C) and three (Figure 1D) applications, with the highest value recorded at the commercial harvest date (day 0) and a drastic reduction during storage. In fruits treated with two applications (Figure 1C), figs treated with OA1 differed significantly from the other treatments: on day 0 they showed the highest ethylene production, whereas by day 10 they showed the lowest. In fruits treated with three applications (Figure 1D), both OA1 and OA2 treatments resulted in significantly lower ethylene production compared with CO on day 10. At harvest (day 0), OA1-treated figs presented the lowest rate.

### 3.2. Physicochemical Parameters

The results for the physicochemical parameters are shown in Table 1. Regarding firmness, no significant differences were found among treatments for either the two- or three-application samples. However, the standard deviation in the fruits treated with two applications was notably high in many cases, which indicates considerable variability in this parameter among fruits. In both cases, a slight loss of firmness was observed during storage. The firmness values of the fruits treated with two applications ranged from 0.49 to 0.74 N mm^−1^, with a mean of 0.62 ± 0.07 N mm^−1^; whereas fruits treated with three applications showed higher firmness, ranging from 0.55 to 1.03 N mm^−1^, with a mean of 0.75 ± 0.12 N mm^−1^.

Concerning TSS, the only significant difference was observed in fruits treated with two applications of OA2, which showed a lower TSS content (18.7 °Brix) than the CO fruits (23.5 °Brix) on day 3 of storage. Overall, TSS values ranged between 18.3 and 23.5 °Brix, with a mean of 20.1 ± 1.2 °Brix.

TA was the physicochemical trait that showed the greatest differences among treatments. Fruits treated with two applications exhibited higher acidity, with values ranging from 0.051 to 0.100% citric acid and a mean of 0.071 ± 0.015% citric acid. The highest values were found on the first two sampling dates (days 0 and 3), with OA1-treated fruit showing significantly higher acidity. TA decreased as storage progressed, and on the final day (day 10), OA2-treated fruit showed the highest acidity. In contrast, fruits treated with three applications showed lower TA and less variability, ranging from 0.048 to 0.070% citric acid, with a mean of 0.060 ± 0.007% citric acid. In this case, OA2-treated fruit consistently showed higher TA throughout storage, although by the end (days 7 and 10) these differences were not significant compared with the control fruit.

With regard to the RI (ripening index; TSS TA^−1^), the general trend was approximately the inverse of that observed for TA. In fruits with two applications, CO samples generally exhibited significantly higher values compared with OA-treated samples. On the other hand, in fruits treated with three applications, the same trend was found on day 0, with CO samples showing a higher RI than the treated ones. However, from day 7 of storage onwards, OA1-treated fruit exhibited the highest RI, while OA2-treated fruit showed the lowest values.

**Figure 2:**
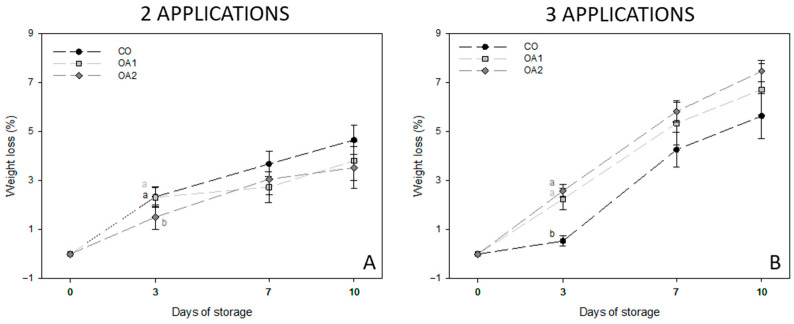
Mean values ± standard deviation of weight loss in fruits treated with two (**A**) and three (**B**) applications at the four sampling dates. Different lowercase letters within each day indicate significant differences (*p* < 0.05) according to Tukey’s test. CO: control; OA1: oxalic acid 1 mM; OA2: oxalic acid 2 mM.

With respect to weight loss (Figure 2), different patterns were observed in fruits treated with two and three applications. In fruits treated with two applications (Figure 2A), the OA treatments resulted in slightly lower weight losses than the control during cold storage. Nevertheless, in fruits treated with three applications (Figure 2B), the opposite trend was found, with CO fruit showing significantly lower weight loss than treated fruit at day 3. However, no significant differences were found at the end of storage, being the CO-treated fruit the lowest value. Furthermore, weight losses in fruits treated with three applications were greater than in those treated with two.

Finally, regarding colour, no clear trends or significant differences were observed among treatments (Table S1). For skin colour, the mean values for L*, C*, and h* were 70.1 ± 1.4, 57.9 ± 0.6, and 102.0 ± 0.8 for fruits treated with two applications, and 68.4 ± 1.2, 60.4 ± 0.6, and 103.8 ± 0.9 for those treated with three applications. These values indicate that the figs showed a yellow-greenish colour, with high brightness and moderate saturation. With respect to flesh colour, all three parameters were lower in both cases, with mean values of 54.1 ± 2.7, 25.8 ± 1.1, and 79.0 ± 1.5 for fruits treated with two applications, and 54.6 ± 2.0, 26.2 ± 1.1, and 78.6 ± 0.8 for those treated with three applications. In this case, the flesh colour corresponded to a yellow-orange tone, less saturated and less luminous.

### 3.3. Profiles of Individual Sugars and Organic Acids

The concentrations of sugars and organic acids are shown in Table 2. For sugars, the levels of glucose, fructose and sucrose were measured, with glucose and fructose being predominant. In figs treated with two applications, glucose ranged from 66.8 to 96.0 g kg^−1^ FW, while fructose varied from 64.7 to 93.8 g kg^−1^ FW. Both reducing sugars showed a similar trend, with no significant differences among treatments at harvest (day 0). However, on subsequent storage days, treated fruit showed lower concentrations of these sugars compared with CO. Sucrose, by contrast, was present at very low levels (0.0–1.5 g kg^−1^ FW).

In fruit treated with three applications, the sugar profile varied slightly. Glucose and fructose remained the predominant sugars and followed a similar trend, although their concentrations showed a wider range of variability (42.8–110.4 and 41.2–105.3 g kg^−1^ FW, respectively). At harvest (day 0), CO showed significantly higher values than the treated fruit. However, on subsequent sampling days, this pattern was reversed, with OA1- and OA2-treated fruit showing significantly higher concentrations of these sugars. Notably, the highest values (110.4 and 105.3 g kg^−1^ FW, respectively) were recorded in OA2-treated fruit on day 3 of cold storage. At the end of storage, this treatment again showed the highest concentrations. As before, sucrose was present at much lower levels (0.0–4.2 g kg^−1^ FW). Significant differences were only observed on day 3, when OA2-treated fruit presented a significantly higher sucrose concentration (4.2 g kg^−1^ FW), coinciding with the highest glucose and fructose levels.

The organic acid profile was determined from the concentrations of oxalic, citric, malic, and succinic acids. In fruit treated with two and three applications, malic acid was the predominant component, with concentrations ranging from 5.4 to 12.1 and from 3.2 to 9.1 g kg^−1^ FW, respectively. Succinic acid was the next most abundant, with concentrations between 2.9 and 6.6 g kg^−1^ FW in fruit treated with two applications, and between 2.4 and 4.24 g kg^−1^ FW in those treated with three. Citric and oxalic acids showed at much lower concentrations, ranging from 0.33 to 1.48 and from 0.04 to 0.179 g kg^−1^ FW, respectively.

In fruit treated with two applications, an increase in all organic acids was observed on day 3 of cold storage. Overall, the OA2 treatment generally resulted in the lowest or among the lowest concentrations across all acids and sampling days. In fruit treated with three applications, a general increase in concentrations was again observed on day 3. On day 7, the OA1 treatment generally showed higher concentrations than both CO and OA2, whereas on day 10, its values were similar to the control.

### 3.4. Enzymatic and Non-Enzymatic Antioxidant Activities

**Figure 3:**
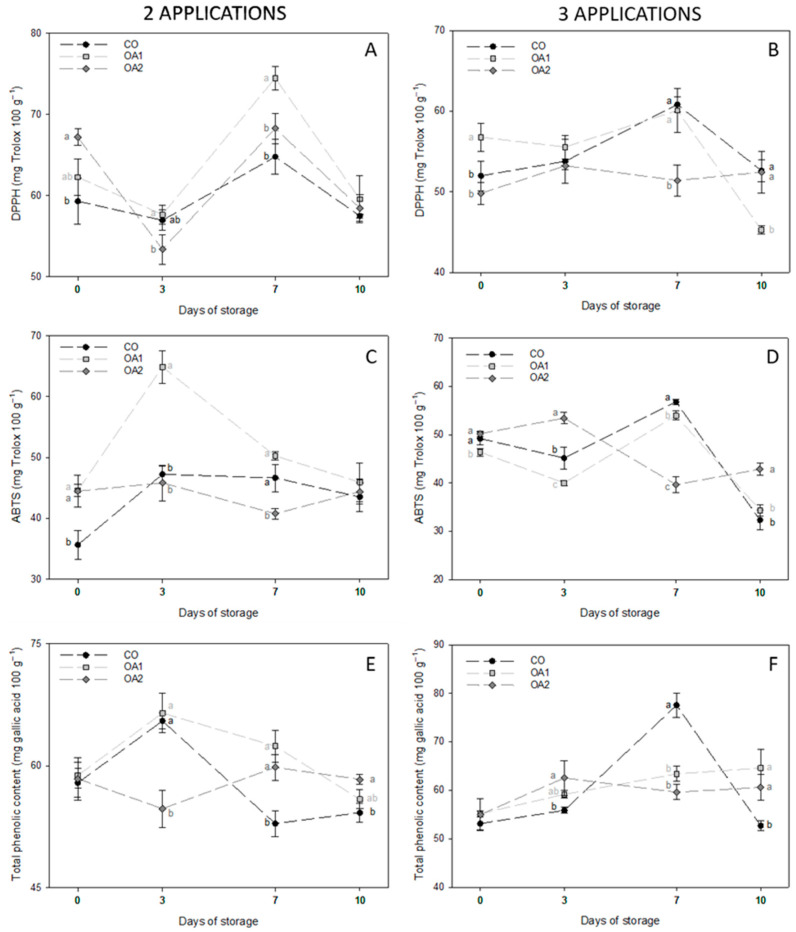
Mean values ± standard deviation of DPPH (**A**,**B**), ABTS (**C**,**D**) and TPC (**E**,**F**) in fruits treated with two and three applications, respectively, at the four sampling dates. Different lowercase letters within each day indicate significant differences (*p* < 0.05) according to Tukey’s test. CO: control; OA1: oxalic acid 1 mM; OA2: oxalic acid 2 mM.

The non-enzymatic antioxidant activity of fruits treated with two and three applications was analysed at the four sampling dates, and the results are shown in Figure 3. DPPH values followed a similar trend in both treatment groups (Figure 3A,B), with an increase in antioxidant activity on day 7 of cold storage, followed by a decline on day 10, resulting in final values comparable to those recorded at day 0. In fruits treated with two applications (Figure 3A), OA1-treated samples exhibited higher antioxidant activity, although no significant differences were observed at the end of storage. In fruits treated with three applications (Figure 3B), OA1-treated fruit showed the highest antioxidant activity at the beginning of storage but declined to the lowest level by the final sampling day. In contrast, OA2-treated fruit showed an increase on day 7, similar to the control fruit.

Regarding the antioxidant activity measured by the ABTS method, differences were found between fruits treated with two and three applications. In fruits treated with two applications (Figure 3C), OA1-treated fruit exhibited the highest antioxidant activity throughout storage, whereas OA2-treated fruit consistently showed among the lowest values. However, by the end of storage, no differences were detected among treatments. In fruits treated with three applications (Figure 3D), CO- and OA1-treated fruit displayed a similar trend, with an increase in antioxidant activity on day 7, followed by a decline on day 10, as observed with DPPH (Figure 3B). In contrast, OA2-treated fruit showed the opposite behaviour, with the lowest activity on day 7 but the highest at the end of storage.

Regarding total phenolic content, fruit treated with two applications of CO and OA1 followed a similar trend, with an increase on day 3 of storage, followed by a gradual decline. On the other hand, OA2-treated fruit showed the opposite pattern, with the lowest TPC observed on day 3, but reaching a significantly higher value than CO by the end of storage (Figure 3E). In fruits treated with three applications (Figure 3F), CO exhibited a significant increase on day 7, before declining to a significantly lower TPC than the treated fruits at the end of storage.

**Figure 4:**
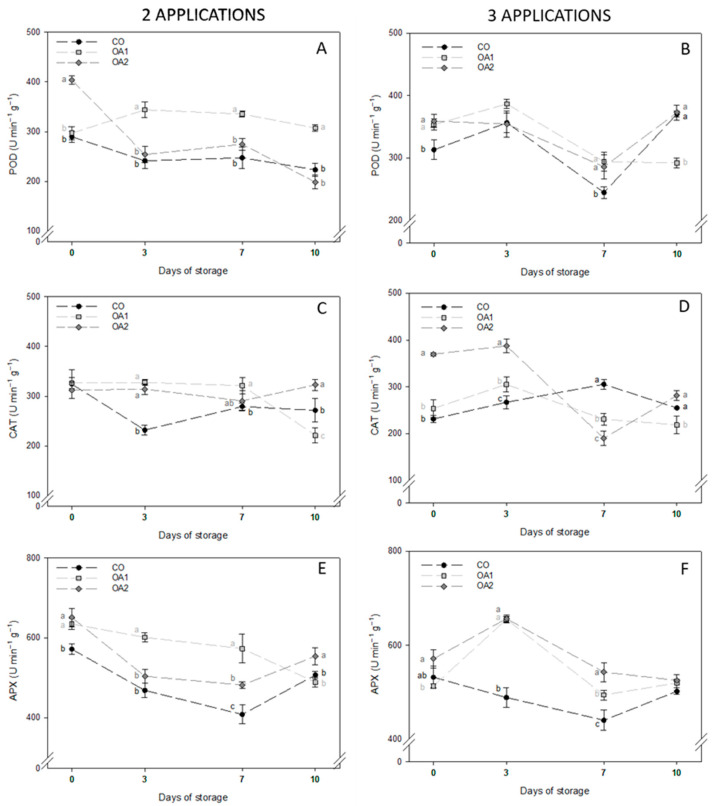
Mean values ± standard deviation of POD (**A**,**B**), CAT (**C**,**D**) and APX (**E**,**F**) activities in fruits treated with two and three applications, respectively, at the four sampling dates. Different lowercase letters within each day indicate significant differences (*p* < 0.05) according to Tukey’s test. APX: ascorbate peroxidase; CAT: catalase; CO: control; OA1: oxalic acid 1 mM; OA2: oxalic acid 2 mM; POD: peroxidase.

Bearing in mind the enzymatic antioxidant activity, the results are shown in Figure 4. Considering POD, different patterns can be observed in fruits treated with two (Figure 4A) and three (Figure 4B) applications. In the first case (Figure 4A), POD activity in CO- and OA2-treated fruit declined from harvest to the end of storage, this decrease being more pronounced in the latter. By contrast, OA1-treated fruit maintained more stable values throughout storage, exhibiting the highest enzymatic activity after 10 days of cold storage. In fruits treated with three applications (Figure 4B), however, OA1-treated fruit showed a decline in POD activity over the storage period, ending with a significantly lower value than CO and OA2-fruit.

Regarding CAT activity, fruits treated with two applications (Figure 4C) showed fewer changes throughout storage than observed for POD. Notably, OA1-treated fruit exhibited the highest enzymatic activity up to day 7 but ultimately showed the lowest value on day 10. In contrast, OA2-treated fruit maintained relatively stable values throughout storage, ending with the highest CAT-activity. For fruits treated with three applications (Figure 4D), CO- and OA1-treated fruit remained approximately steady throughout storage, whereas OA2-treated fruit began with the highest activity but reached a significant minimum on day 7. By the end of storage, however, OA2-treated fruit exhibited greater antioxidant activity than OA1-treated fruit.

Finally, regarding APX enzymatic activity, in fruits treated with two applications (Figure 4E), CO- and OA2-treated fruit showed a similar trend, with activity decreasing until day 7, followed by a slight increase on day 10, with OA2-treated fruit showing the highest APX activity. In contrast, OA1-treated fruit maintained a general decline in activity until day 10. In fruits treated with three applications (Figure 4F), a clear difference was observed between CO-treated fruit, which showed a decrease in activity on day 3 followed by a recovery, and OA-treated fruit, which exhibited a significant rise on day 3 followed by a decrease until day 10. Nevertheless, no differences were found among treatments on the final day.

### 3.5. Assessment of Membrane Lipid Oxidation

**Figure 5:**
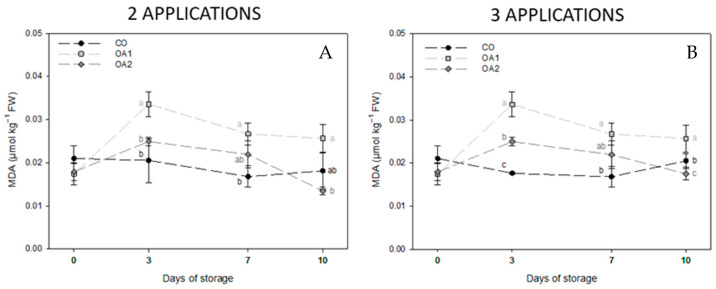
Mean values ± standard deviation of MDA in fruits from tree treated with two (**A**) and three (**B**) applications, respectively, at the four sampling dates. Different lowercase letters within each day indicate significant differences (*p* < 0.05) according to Tukey’s test. CO: control; FW: fresh weight; MDA: malondialdehyde; OA1: oxalic acid 1 mM; OA2: oxalic acid 2 mM.

Membrane oxidation, expressed as MDA concentration, is shown in Figure 5. In fruits from trees treated with two applications (Figure 5A), OA-treated fruit showed higher levels of oxidation throughout storage, particularly OA1-fruit. By the final sampling date, differences between the two OA treatments were observed, although neither differed significantly from CO-fruit. For fruits from trees treated with three applications (Figure 5B), a similar trend was observed, with treated fruit showing greater oxidation than CO-fruit during the intermediate storage period. However, on day 10, OA2-treated fruit displayed the lowest MDA concentration with no significant differences respect to CO treatment.

## 4. Discussion

Although figs are generally classified as climacteric fruits—characterised by moderate respiratory and ethylene production rates—certain tissues of the syconium also exhibit non-climacteric behaviour. This means they must be harvested when almost fully ripe to ensure optimal quality [10,18,38]. Furthermore, varietal differences can lead to variations in the fruit’s physiological behaviour during cold storage. Moreover, figs ripen gradually from the bottom to the top of the same branch [30], which makes it more challenging to harvest them at their optimum ripeness. In general, green-skinned figs show a shelf life of around 5 days under cold conditions [39].

In our study, the respiration rate decreased during storage in fruits subjected to two applications, where the OA2 treatment significantly reduced this parameter. By contrast, figs treated with three applications of OA showed a significantly higher respiration rate than the control. These results contradict the findings of other authors in lemon and pomegranate, where OA-treated fruit with 0.1, 0.5, 1, 5, and 10 mM showed a decrease in respiration rate [26,40]. However, other studies have reported an increase in respiration rate at specific storage sampling points in OA-treated fruits, such as apricots and artichokes [14,41]. Our values were higher than those reported by other authors for green figs (cv. Cuello Dama Blanco), which showed mean values of 25.07 mL CO_2_ kg^−1^ h^−1^ [38]. Conversely, the ethylene production rates obtained in our study were also higher than those reported for other varieties (0.72–1.78 µL C_2_H_4_ kg^−1^ h^−1^ in green varieties) [10]. Nevertheless, the rate of ethylene production decreased in treated figs, showing a pattern similar to that reported for other OA-treated fruits [14,29].

Among the most important parameters determining fruit quality are those related to morphological and sensory traits, such as firmness, TSS, TA, RI and colour. Measuring and controlling these traits is essential to ensure that the fruit is harvested at its optimum quality stage [10,42]. Moreover, appearance is the primary characteristic influencing consumer acceptance [7]. In the present study, preharvest application of OA did not result in significant changes in firmness or TSS.

Firmness is generally directly related to the degree of ripening or senescence during the postharvest life, although its values vary significantly depending on the cultivar [12]. In our study, the values obtained were similar to those reported by other authors (0.75–0.90 N mm^−1^) [42]. A slight decrease in firmness during storage has also been described by other authors [4], who attributed it to changes in cell structure integrity.

TSS and TA are closely related to consumer acceptance, with sweeter fruits exhibiting lower acidity and being generally preferred [10,43]. Other green fig varieties, such as cv. Cuello Dama Blanco, have shown TSS values ranging from 19.1 to 24.37 °Brix [4,10,38,42], similar to the values obtained in our study. Regarding TA, variations in acidity levels can affect overall flavour perception and influence consumer preference. TA is variety-dependent, with differences arising from both genetic traits and ecological factors. Previous studies have reported TA values between 0.07 and 0.35% [4,5,38,42]. In our trial, cv. Calabacita figs exhibited slightly lower TA values and a modest decrease during storage, consistent with fig maturation during this period. However, it was found that OA2 treatment maintained significantly higher acidity levels at the end of storage for both two- and three-application treatments, as previously observed in OA-treated lemons [40]. With respect to the ripening index (RI; TSS TA^−1^), an increase was observed throughout storage, mainly due to the loss of TA. Differences were found between the two- and three-application treatments, with values ranging from 202.6 to 368.6 in the two-application treatment and from 275.8 to 417.8 in the three-application treatment. Figs subjected to three applications showed a higher RI and, potentially, greater consumer acceptability. Depending on the species, OA has shown varying effects on RI. In pomegranate, it markedly affected both TSS and TA, and consequently the RI [26]. In contrast, in plum, this impact was less pronounced, as a reduction in TA was also observed during storage—similar to the findings of the present study. Nevertheless, OA-treated plums exhibited a higher RI and, therefore, delayed senescence [29]. This result is consistent with the behaviour observed in figs treated with three applications after storage. Finally, in strawberry, an increase in RI and greater sweetness were reported [44]. These findings highlight that exogenous OA application influences the metabolic pathways associated with fruit ripening. With regard to weight loss, the effect depended more on the number of applications than on the concentration used, with two applications reducing weight loss. This effect was not observed with three applications. Studies conducted on lemons have reported reductions in weight loss of up to 40% compared with untreated fruit [40]. This is likely related to the ability of OA to reinforce cell walls, thereby maintaining better firmness and extending the product’s shelf life.

Colour is one of the final physicochemical traits most closely associated with quality and consumer acceptance, as it is strongly linked to the ripening stage and the fruit’s bioactive profile [4,6,10]. The treated figs showed hue values like those reported by Vieira et al. [45] for green-skinned figs from Portugal, but higher luminosity and chroma values than those observed by De Bruno et al. [6]. In our study, preharvest application of OA did not affect fig colour parameters, although other authors have reported changes in apricot colour characteristics [14].

The presence and profile of reducing sugars affects the flavour of figs and, consequently, consumer acceptability [4,10]. The relative amount of these constituents depends on the fruit’s metabolic activity and is influenced by genetic factors, ripening and storage, and storage condition [4]. Moreover, glucose and fructose have been shown to play an important role in protecting plants against oxidative stress [23]. In figs, the main sugars are glucose (70.8–210.0 g kg^−1^ FW) and fructose (43.3–90.9 g kg^−1^ FW), followed by sucrose (0.2–3.8 g kg^−1^ FW), which is present in much lower amounts [3,4,8], in agreement with the data obtained in our study. The increase in glucose and fructose concentrations in fruit treated with three OA applications is consistent with the observations reported by Wang et al. [23] in apricot.

Organic acids are primary metabolites present in all plants but particularly abundant in fruits. They possess antioxidant properties, and their content and composition influence flavour development. Several authors have identified malic acid as the predominant one, accounting for 56–96% of the total organic acid content. Other acids commonly detected in figs include citric, succinic and oxalic acids [4,46,47]. In our samples, the organic acid profile corresponded with these findings, as well as with the concentrations reported by other authors [3,4,47].

Regarding the bioactive profile, figs are fruit with well-recognised antioxidant activity, which benefits both the fruit itself—by providing protection against various biotic and abiotic stresses—and consumers, as these compounds confer health-promoting effects, such as anti-inflammatory or antibacterial activity [6,10,11]. The profile of bioactive compounds influences flavour and aroma and is strongly affected by genotype, ripening stage and other agronomic factors (i.e., soil conditions) [1,6]. In our study, non-enzymatic antioxidant activity and TPC followed a similar trend, showing values within the range reported by other authors for green-skinned fig varieties [1,6]. Overall, OA-treated figs exhibited higher antioxidant activity and TPC at the end of storage, particularly in the case of OA2-treated fruit. These findings are in agreement with those reported in lemon and kiwifruit [24,40], where OA treatments promoted the accumulation of phenolic compounds during cold storage through activation of the phenylalanine ammonium lyase (PAL) enzyme, a key component of the phenylpropanoid pathway and a precursor of many plant metabolites.

On the other hand, fruit protection systems also include the enzymatic mechanisms of POD, CAT and APX, which play an important role in eliminating oxidative stress markers such as MDA or H_2_O_2_. Some authors have reported that CAT exhibits the highest enzymatic activity in figs [10]; however, in our study, APX showed the greatest activity. A similar pattern was observed for all three enzymes, as the treatments maintained or enhanced enzymatic activity compared with the control at the end of cold storage, especially in the OA2 treatment. These findings are consistent with those reported by other authors in pomegranate [26], grape [48], lemon [40] or blueberry [27], where preharvest OA treatments stimulated antioxidant enzyme activity to counteract oxidative stress during cold storage.

Finally, the oxidative stress marker MDA serves as an indicator of senescence, loss of cell membrane integrity, and fruit damage [10]. Several authors have reported that preharvest OA application can help delay fruit senescence by reducing cell membrane degradation and, consequently, MDA accumulation [23,27,49]. Our results support this observation, since although OA-treated fruit showed slightly higher MDA values during storage, by day 10, the OA2 treatment in both two- and three-application groups showed the lowest MDA concentrations, with no significant differences in the two-application group, indicating better preservation of fruit integrity. This outcome is consistent with the higher enzymatic activity values observed at the same time point. Therefore, we can conclude that two preharvest applications with OA 2 mM extend the storability of green-skinned figs to acceptable commercial quality under cold storage for up to 10 days.

## 5. Conclusions

The preharvest application of oxalic acid exerts a significant multifaceted influence on the postharvest life of ‘Calabacita’ figs, with the number of applications emerging as a critical factor. The results indicate that two applications are more effective than three in extending postharvest cold storage.

Specifically, two applications of OA successfully limited weight loss, reduced the respiration rate, and lowered ethylene production. In contrast, three applications, although enhancing the sugar profile, had an adverse effect by increasing both weight loss and respiration.

A consistent benefit across all OA treatments was the stimulation of the fruit’s antioxidant system. OA treatment, particularly at a concentration of 2 mM (OA2), maintained higher titratable acidity and promoted greater total phenolic content, alongside enhanced enzymatic and non-enzymatic antioxidant activity. This enhanced oxidative protection was further supported by lower MDA concentrations at the end of cold storage, indicating better membrane integrity and delayed senescence.

In conclusion, while the beneficial role of preharvest OA application in figs has been confirmed, our results clearly demonstrate that two applications at 2 mM represent the most effective strategy. Implementing this practice may serve as a valuable tool for maintaining fruit quality and extending the postharvest life of fresh figs.

## Figures and Tables

**Table 1:** Mean values ± standard deviation of the physicochemical parameters analysed at the four sampling dates. Different lowercase letters within each day and number of applications indicate significant differences (*p* < 0.05) according to Tukey’s test. CO: control; OA1: oxalic acid 1 mM; OA2: oxalic acid 2 mM; RI: ripening index; TA: titratable acidity; Trt: treatment; TSS: total soluble solids.

	Day	Trt	Firmness (N mm^−1^)	TSS (°Brix)	TA (% Citric Acid)	RI (TSS TA^−1^)
2 APPLICATIONS	0	CO	0.63 ± 0.19	20.7 ± 2.9	0.071 ^b^ ± 0.003	279.1 ^a^ ± 13.2
		OA1	0.63 ± 0.09	19.9 ± 1.4	0.083 ^a^ ± 0.006	224.5 ^b^ ± 8.5
		OA2	0.62 ± 0.16	20.2 ± 1.0	0.072 ^b^ ± 0.002	279.3 ^a^ ± 11.6
	3	CO	0.62 ± 0.16	23.5 ^a^ ± 2.2	0.079 ^b^ ± 0.001	288.4 ^a^ ± 13.3
		OA1	0.70 ± 0.14	21.5 ^ab^ ± 0.7	0.100 ^a^ ± 0.005	206.2 ^b^ ± 2.1
		OA2	0.63 ± 0.06	18.7 ^b^ ± 1.0	0.087 ^b^ ± 0.005	202.6 ^b^ ± 4.5
	7	CO	0.68 ± 0.11	18.9 ± 0.5	0.055 ± 0.002	342.0 ^a^ ± 16.7
		OA1	0.74 ± 0.06	19.9 ± 0.5	0.068 ± 0.007	289.2 ^b^ ± 16.6
		OA2	0.64 ± 0.10	19.0 ± 0.6	0.065 ± 0.008	280.4 ^b^ ± 21.1
	10	CO	0.49 ± 0.03	19.5 ± 1.1	0.051 ^b^ ± 0.003	368.6 ^a^ ± 10.3
		OA1	0.52 ± 0.02	20.1 ± 0.6	0.055 ^b^ ± 0.001	367.7 ^a^ ± 9.1
		OA2	0.53 ± 0.02	20.7 ± 0.5	0.061 ^a^ ± 0.002	341.3 ^b^ ± 4.2
3 APPLICATIONS	0	CO	0.84 ± 0.02	21.0 ± 0.1	0.061 ^b^ ± 0.002	342.7 ^a^ ± 11.3
		OA1	0.83 ± 0.10	18.3 ± 1.4	0.066 ^ab^ ± 0.001	275.8 ^b^ ± 19.2
		OA2	0.74 ± 0.14	19.9 ± 1.8	0.070 ^a^ ± 0.003	272.3 ^b^ ± 10.2
	3	CO	0.78 ± 0.07	18.9 ± 0.9	0.057 ^b^ ± 0.002	330.9 ± 28.1
		OA1	0.70 ± 0.11	21.4 ± 1.5	0.060 ^b^ ± 0.003	341.9 ± 10.9
		OA2	0.72 ± 0.04	20.8 ± 0.2	0.069 ^a^ ± 0.002	300.1 ± 8.7
	7	CO	0.62 ± 0.05	19.4 ± 1.0	0.059 ^ab^ ± 0.003	342.8 ^b^ ± 10.9
		OA1	0.55 ± 0.06	21.3 ± 1.1	0.056 ^b^ ± 0.005	408.2 ^a^ ± 16.2
		OA2	0.63 ± 0.02	20.3 ± 0.8	0.066 ^a^ ± 0.004	295.3 ^c^ ± 9.8
	10	CO	0.76 ± 0.12	19.4 ± 0.6	0.053 ^ab^ ± 0.001	375.3 ^b^ ± 8.5
		OA1	0.77 ± 0.05	21.3 ± 2.2	0.048 ^b^ ± 0.003	417.8 ^a^ ± 23.8
		OA2	1.03 ± 0.25	18.4 ± 1.6	0.059 ^a^ ± 0.003	337.9 ^c^ ± 37.0

**Table 2:** Mean values ± standard deviation of sugars and organic acids quantified in the treated figs. Different lowercase letters within each day and number of applications indicate significant differences (*p* < 0.05) according to Tukey’s test. CO: control; FW: fresh weight; OA1: oxalic acid 1 mM; OA2: oxalic acid 2 mM; Trt: treatment.

	Day	Trt	Sugars (g kg^−1^ FW)			Organic Acids (g kg^−1^ FW)			
			Glucose	Fructose	Sucrose	Oxalic Acid	Citric Acid	Malic Acid	Succinic Acid
2 APPLICATIONS	0	CO	77.7 ± 1.7	75.4 ± 1.5	0.38 ^ab^ ± 0.66	0.11 ^a^ ± 0.01	0.87 ^b^ ± 0.04	7.3 ^ab^ ± 0.1	4.5 ± 0.2
		OA1	80.4 ± 4.1	77.3 ± 8.9	1.5 ^a^ ± 0.4	0.11 ^a^ ± 0.03	1.14 ^a^ ± 0.05	7.7 ^a^ ± 0.2	4.5 ± 0.3
		OA2	75.3 ± 9.4	73.3 ± 4.5	0.00 ^b^ ± 0.00	0.09 ^b^ ± 0.07	0.80 ^b^ ± 0.04	7.1 ^b^ ± 0.3	4.4 ± 0.1
	3	CO	96.0 ^a^ ± 3.4	93.8 ^a^ ± 3.6	0.00 ± 0.00	0.157 ^b^ ± 0.003	1.32 ^ab^ ± 0.07	12.1 ^a^ ± 0.6	6.6 ^a^ ± 0.3
		OA1	89.5 ^ab^ ± 2.6	85.9 ^ab^ ± 3.8	0.06 ± 0.09	0.179 ^a^ ± 0.007	1.48 ^a^ ± 0.03	10.4 ^b^ ± 0.4	5.5 ^b^ ± 0.3
		OA2	79.4 ^b^ ± 6.3	77.2 ^b^ ± 5.9	0.00 ± 0.00	0.153 ^b^ ± 0.003	1.19 ^b^ ± 0.09	9.5 ^b^ ± 0.2	4.7 ^c^ ± 0.2
	7	CO	86.1 ^a^ ± 3.4	83.9 ^a^ ± 3.4	0.21 ± 0.37	0.105 ^a^ ± 0.004	0.74 ^b^ ± 0.02	6.87 ± 0.09	3.3 ± 0.2
		OA1	69.0 ^b^ ± 4.7	66.5 ^b^ ± 4.0	0.18 ± 0.32	0.10 ^a^ ± 0.01	0.91 ^a^ ± 0.02	6.9 ± 0.2	3.5 ± 0.4
		OA2	68.7 ^b^ ± 6.2	67.1 ^b^ ± 6.0	0.00 ± 0.00	0.064 ^b^ ± 0.005	0.80 ^ab^ ± 0.09	6.6 ± 0.4	3.5 ± 0.1
	10	CO	85.4 ^a^ ± 3.9	81.3 ^a^ ± 8.5	0.00 ± 0.00	0.095 ^a^ ± 0.007	0.87 ^a^ ± 0.05	7.1 ^a^ ± 0.2	3.5 ± 0.2
		OA1	69.0 ^b^ ± 2.3	68.3 ^b^ ± 2.4	0.00 ± 0.00	0.085 ^ab^ ± 0.005	0.68 ^b^ ± 0.04	6.5 ^a^ ± 0.2	3.2 ± 0.3
		OA2	66.8 ^b^ ± 1.6	64.7 ^b^ ± 1.6	0.28 ± 0.49	0.077 ^b^ ± 0.005	0.68 ^b^ ± 0.02	5.4 ^b^ ± 0.3	2.9 ± 0.3
3 APPLICATIONS	0	CO	91.1 ^a^ ± 5.5	88.3 ^a^ ± 5.1	0.78 ± 1.4	0.044 ± 0.004	0.49 ^a^ ± 0.02	5.1 ^a^ ± 0.3	3.5 ^a^ ± 0.3
		OA1	42.8 ^b^ ± 5.1	41.2 ^b^ ± 4.7	0.00 ± 0.00	0.047 ± 0.008	0.33 ^b^ ± 0.02	3.2 ^b^ ± 0.3	2.4 ^b^ ± 0.3
		OA2	50.7 ^b^ ± 2.4	48.7 ^b^ ± 2.6	0.29 ± 0.50	0.040 ± 0.007	0.46 ^a^ ± 0.02	4.8 ^a^ ± 0.2	2.5 ^b^ ± 0.4
	3	CO	80.2 ^c^ ± 5.9	77.3 ^b^ ± 6.0	0.57 ^b^ ± 0.49	0.069 ^c^ ± 0.003	0.66 ^b^ ± 0.03	7.88 ^b^ ± 0.06	4.2 ± 0.1
		OA1	98.8 ^b^ ± 1.1	96.4 ^a^ ± 1.1	0.00 ^b^ ± 0.00	0.13 ^a^ ± 0.01	0.858 ^a^ ± 0.002	9.1 ^a^ ± 0.1	4.07 ± 0.04
		OA2	110.4 ^a^ ± 2.6	105.3 ^a^ ± 2.4	4.2 ^a^ ± 0.9	0.090 ^b^ ± 0.004	0.67 ^b^ ± 0.02	7.7 ^b^ ± 0.4	4.24 ± 0.05
	7	CO	66.7 ^b^ ± 3.5	64.1 ^b^ ± 3.9	0.10 ± 0.17	0.050 ^c^ ± 0.001	0.53 ^b^ ± 0.04	6.5 ^b^ ± 0.2	2.9 ^b^ ± 0.2
		OA1	81.3 ^a^ ± 7.6	78.7 ^a^ ± 7.3	0.26 ± 0.45	0.106 ^a^ ± 0.007	0.635 ^a^ ± 0.002	7.9 ^a^ ± 0.1	3.7 ^b^ ± 0.3
		OA2	66.3 ^b^ ± 1.8	64.0 ^b^ ± 2.0	0.27 ± 0.23	0.069 ^b^ ± 0.004	0.56 ^b^ ± 0.03	6.6 ^b^ ± 0.3	3.4 ^ab^ ± 0.2
	10	CO	64.3 ^b^ ± 3.3	62.0 ^b^ ± 2.8	0.64 ± 0.11	0.064 ^a^ ± 0.003	0.56 ^a^ ± 0.03	6.6 ^a^ ± 0.2	3.6 ^a^ ± 0.2
		OA1	69.5 ^ab^ ± 3.1	66.6 ^ab^ ± 3.2	0.95 ± 1.26	0.063 ^a^ ± 0.003	0.52 ^a^ ± 0.02	7.0 ^a^ ± 0.2	3.5 ^a^ ± 0.3
		OA2	73.5 ^a^ ± 3.5	70.8 ^a^ ± 2.7	0.00 ± 0.00	0.041 ^b^ ± 0.003	0.44 ^b^ ± 0.04	5.5 ^b^ ± 0.3	2.84 ^b^ ± 0.09

## Data Availability

The raw data supporting the conclusions of this article will be made available by the authors on request.

## Supplementary Materials

The following supporting information can be downloaded at: https://www.mdpi.com/article/10.3390/foods14234061/s1; Table S1: Mean values ± standard deviation skin and flesh colour parameters measured in the treated figs at the four sampling dates. Different lowercase letter in each day and number of applications indicates significant differences (*p* < 0.05) using Tukey test. CO: control; AO1: oxalic acid 1 mM; AO2: oxalic acid 2 mM.

## Abbreviations

The following abbreviations are used in this manuscript:

ANOVA	One-way analysis of variance
APX	Ascorbate peroxidase
CAT	Catalase
CO	Control
FW	Fresh weight
MDA	Malondialdehyde
PAL	Phenylalanine ammonium lyase
POD	Peroxidase
OA	Oxalic acid
OA1	Oxalic acid 1 mM
OA2	Oxalic acid 2 mM
TA	Titratable acidity
TAA	Total antioxidant activity
TPC	Total phenolic content
Trt	Treatment
TSS	Total soluble solids
RI	Ripening index
