# Supplementary material for: Preharvest Application of Oxalic Acid to ‘Calabacita’ Fresh Figs: Effects on Physicochemical and Antioxidant Profile During Cold Storage
Source: Foods. 2025 Nov 27;14(23):4061. doi: 10.3390/foods14234061 (PMC12691960; doi:10.3390/foods14234061)
Supplement: Supplementary file 1 [file foods-14-04061-s001.zip › foods-3989439-supplementary.pdf]

Table S1. Mean values ± standard deviation skin and flesh color parameters measured in the treated figs at the four sampling dates. Different lowercase letter in each day and number of applications indicates significant differences ($p<0.05$) using Tukey test. CO: control; AO1: oxalic acid 1 mM; AO2: oxalic acid 2 mM.

	Day	Trt	Skin colour			Flesh colour		
			L*	C*	h*	L*	C*	h*
2 APPLICATIONS	0	CO	70.8a±1.1	58.4±0.5	103.7±1.5	56.7±3.3	26.4±1.7	80.7±0.8
		OA1	69.7ab±1.2	57.0±0.8	101.8±0.9	57.3±1.6	27.9±0.6	79.4±1.1
		OA2	67.1b±1.3	57.5±1.0	102.4±1.5	56.3±2.5	25.8±1.3	79.6±0.7
	3	CO	68.3b±0.3	58.8±0.8	101.9±1.7	57.3±2.3	25.6±0.7	81.6a±0.4
		OA1	69.4ab±0.9	58.2±0.4	100.9±1.2	54.3±2.2	27.2±1.0	77.5c±0.2
		OA2	71.1a±1.0	59.1±0.8	101.2±1.7	56.9±0.6	26.6±0.2	79.8b±0.5
	7	CO	71.3±3.1	57.7±0.7	102.2a±2.5	52.8±0.7	24.5ab±0.5	78.2a±0.8
		OA1	70.8±0.1	57.4±0.7	101.6b±0.4	51.8±2.2	25.1a±0.4	75.9b±0.7
		OA2	69.4±1.0	57.7±0.4	102.2a±1.4	52.8±1.1	24.1b±0.3	78.3a±0.4
	10	CO	71.6±0.3	58.1±0.5	101.1±0.4	50.6±0.9	25.4±0.6	78.0±1.0
		OA1	69.9±0.7	57.7±0.7	103.1±0.5	51.2±0.9	25.5±0.3	79.5±0.3
		OA2	71.4±1.0	57.7±0.3	102.3±0.1	51.2±0.5	25.5±0.1	79.0±0.6
3 APPLICATIONS	0	CO	67.4±0.5	59.5±0.4	104.6±1.3	58.5±2.0	27.3±0.8	79.8±0.8
		OA1	69.5±1.8	60.6±0.8	104.6±0.9	56.3±1.2	26.7±0.5	79.1±0.5
		OA2	68.6±1.3	60.2±0.3	103.6±2.0	56.2±0.8	26.2±0.6	79.6±0.3
	3	CO	67.3±2.8	60.5±1.4	104.2±1.9	55.2±1.1	28.5a±0.8	78.3±0.2
		OA1	70.2±0.8	59.6±1.3	102.9±0.6	52.2±2.5	27.1ab±0.8	76.8±0.6
		OA2	67.9±0.7	61.6±0.6	102.9±1.6	53.4±1.9	26.4b±0.3	78.1±1.0
	7	CO	68.4±1.4	60.0±0.3	104.2±1.2	55.4±1.6	25.5±0.3	78.9±1.2
		OA1	70.4±0.9	60.5±1.1	102.8±0.3	53.5±2.1	25.4±0.8	78.1±0.7
		OA2	68.9±0.5	61.2±0.1	102.2±0.7	55.1±1.5	25.2±0.9	78.7±0.6
	10	CO	68.1a±0.3	60.5±0.8	104.7±0.6	53.9±2.9	25.5±0.6	79.5±1.2
		OA1	67.1b±0.6	60.2±0.3	104.6±1.4	51.0±1.8	24.9±0.5	78.1±0.3
		OA2	66.6b±0.2	60.5±1.3	104.3±2.1	54.7±1.6	25.6±0.3	78.2±0.8
